# Correction: Queuine, a bacterial-derived hypermodified nucleobase, shows protection in *in vitro* models of neurodegeneration

**DOI:** 10.1371/journal.pone.0261515

**Published:** 2021-12-13

**Authors:** The PLOS ONE Staff

There are errors in Fig 1. Several of the labels are incorrect. The authors have provided a corrected version here. The publisher apologizes for the errors.

**Fig 1:**
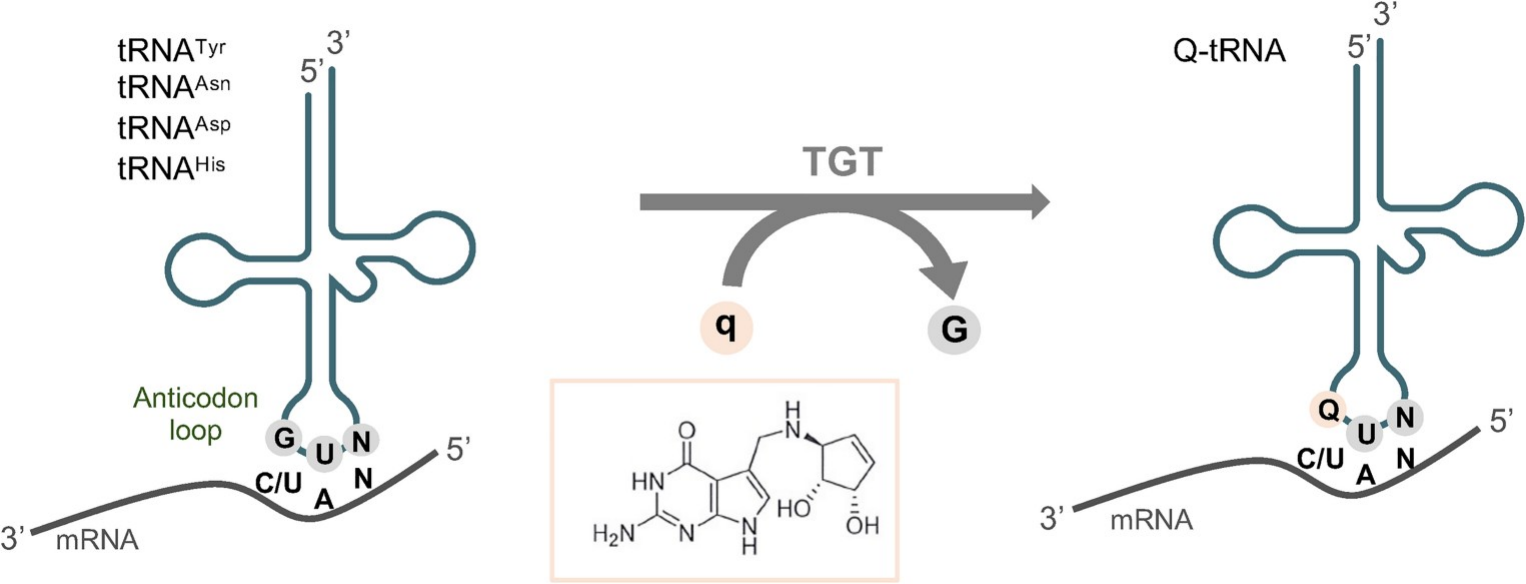
Queuine integration into tRNAs. In eukaryotic cells, the tRNA guanine transglycosylase (TGT) enzyme exchanges guanine (G) with the nucleobase queuine (q) at the wobble position (position 34, first base of the anticodon) of tRNAs that contain a GUN anticodon sequence (N = any base) and are specific to tRNA isoacceptors for tyrosine (tRNA^Tyr^), asparagine (tRNA^Asn^), aspartic acid (tRNA^Asp^) and histidine (tRNA^His^). Queuosine (Q) is the corresponding nucleoside of q and Q-tRNA base-pairing with NAY codons (Y = C or U) impacts speed and fidelity of mRNA translation [14, 20–22].
