# Supplementary material for: Queuine, a bacterial-derived hypermodified nucleobase, shows protection in in vitro models of neurodegeneration
Source: PLoS One. 2021 Aug 11;16(8):e0253216. doi: 10.1371/journal.pone.0253216 (PMC8357117; doi:10.1371/journal.pone.0253216)
Supplement: S1 Appendix — (PDF) [file pone.0253216.s004.pdf]

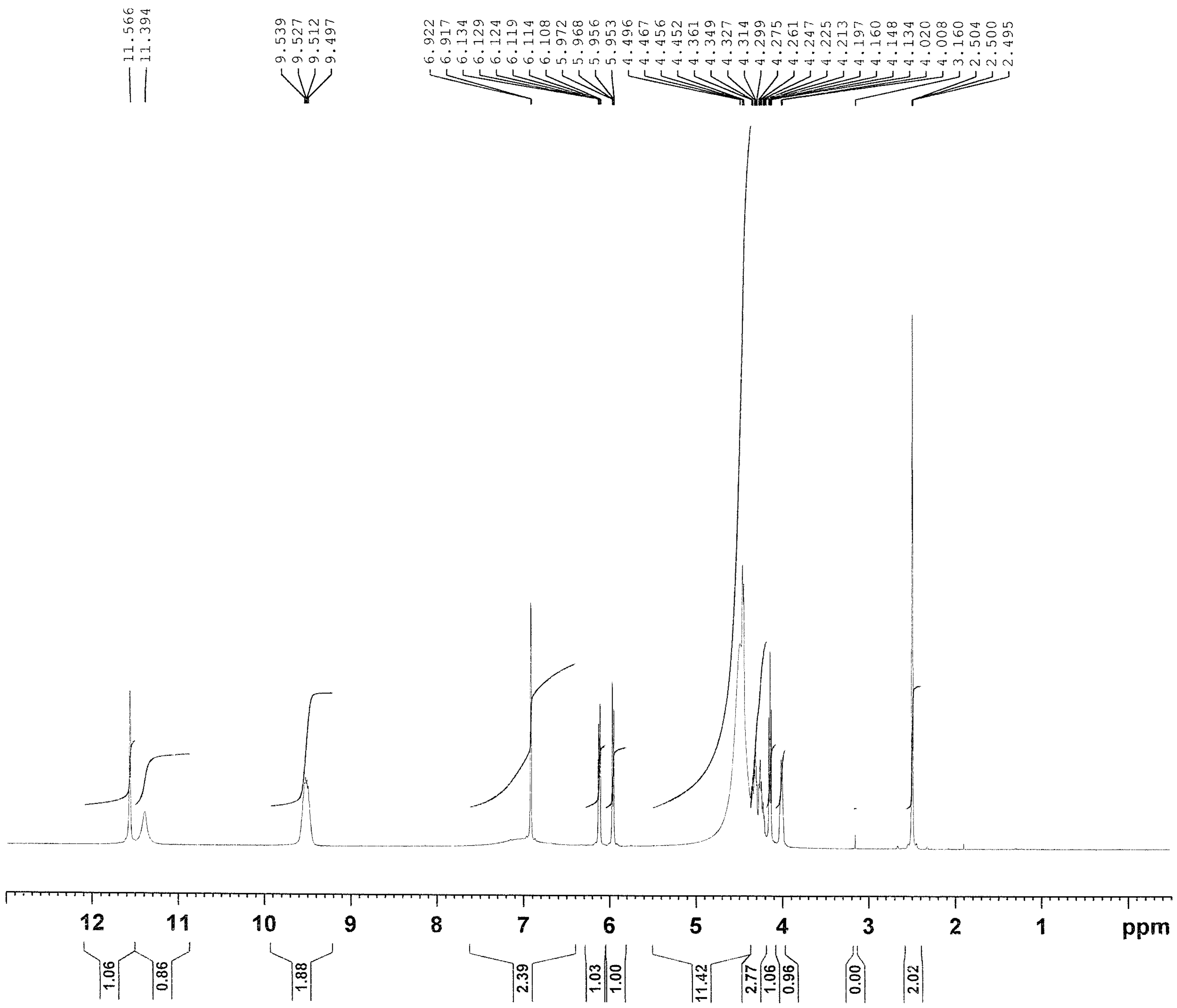


```
Current Data Parameters
NAME        SN0026L5-DMSO
EXPNO                  10
PROCNO                  1

F2 - Acquisition Parameters
Date_            20201006
Time                 9.18
INSTRUM             spect
PROBHD   5 mm PABBO BB/
PULPROG              zg30
TD                  32768
SOLVENT              DMSO
NS                     16
DS                      2
SWH             7978.724 Hz
FIDRES          0.243491 Hz
AQ             2.0534613 sec
RG                 136.69
DW                 62.667 usec
DE                   6.50 usec
TE                  296.2 K
D1            1.00000000 sec
TD0                     1

======== CHANNEL f1 ========
SFO1         399.7524686 MHz
NUC1                   1H
P1                   9.00 usec
PLW1         19.20000076 W

F2 - Processing parameters
SI                  65536
SF           399.7500032 MHz
WDW                    EM
SSB       0
LB                   0.30 Hz
GB        0
PC                   1.00
```

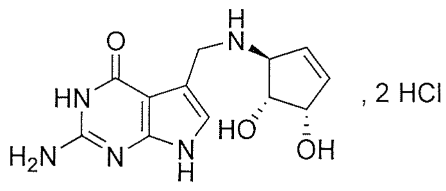

^{1}H NMR (DMSO d6)
Queuine dihydrochloride

^{1}H NMR (DMSO d6)
Queuine dihydrochloride

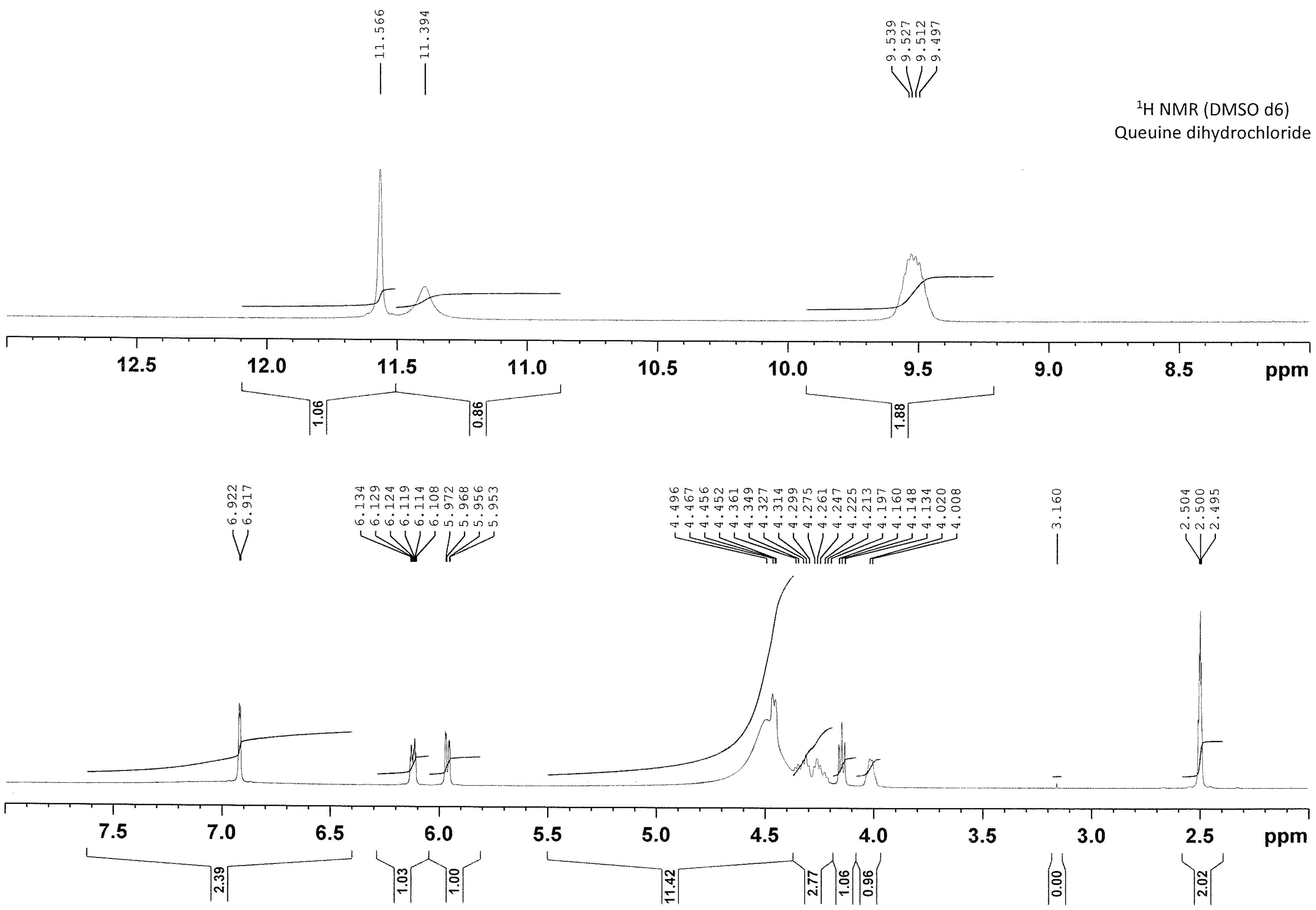

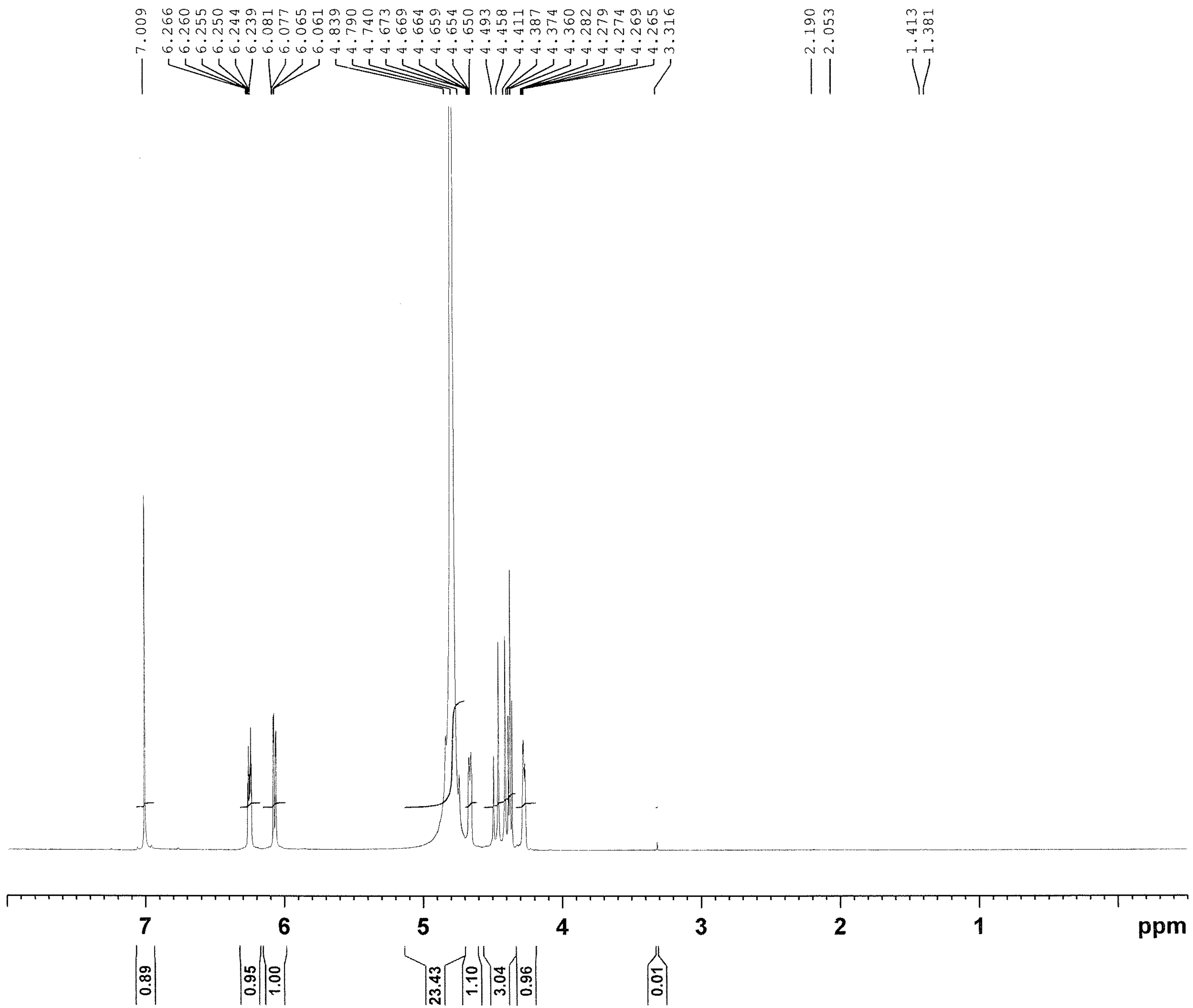


```
Current Data Parameters
NAME         SN0026-L5-D2O
EXPNO                   10
PROCNO                   1

F2 - Acquisition Parameters
Date_             20201005
Time                 11.06
INSTRUM              spect
PROBHD    5 mm PABBO BB/
PULPROG               zg30
TD                   32768
SOLVENT                D2O
NS                      16
DS                       2
SWH              7978.724 Hz
FIDRES           0.243491 Hz
AQ              2.0534613 sec
RG                   61.21
DW                  62.667 usec
DE                    6.50 usec
TE                   296.2 K
D1             1.00000000 sec
TD0                      1

======== CHANNEL f1 ========
SFO1          399.7524686 MHz
NUC1                    1H
P1                    9.00 usec
PLW1          19.20000076 W

F2 - Processing parameters
SI                   65536
SF            399.7499654 MHz
WDW                     EM
SSB       0
LB                    0.30 Hz
GB        0
PC                    1.00
```

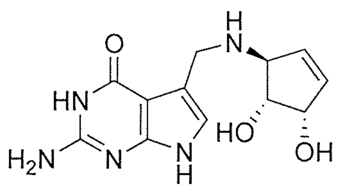

^{1}H NMR (D_2O)

Queuine dihydrochloride

^1H NMR (D_2O)
Queuine dihydrochloride

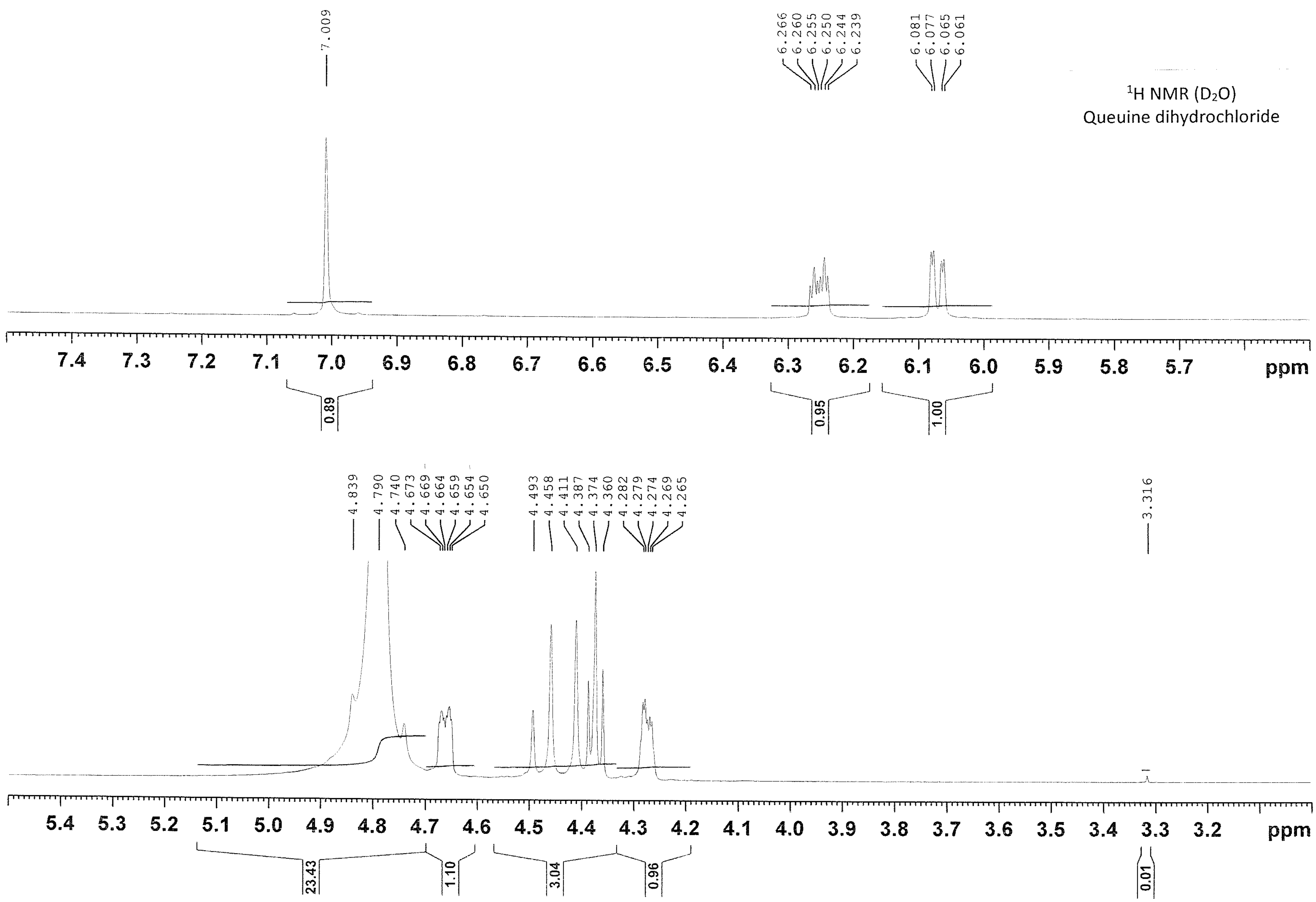

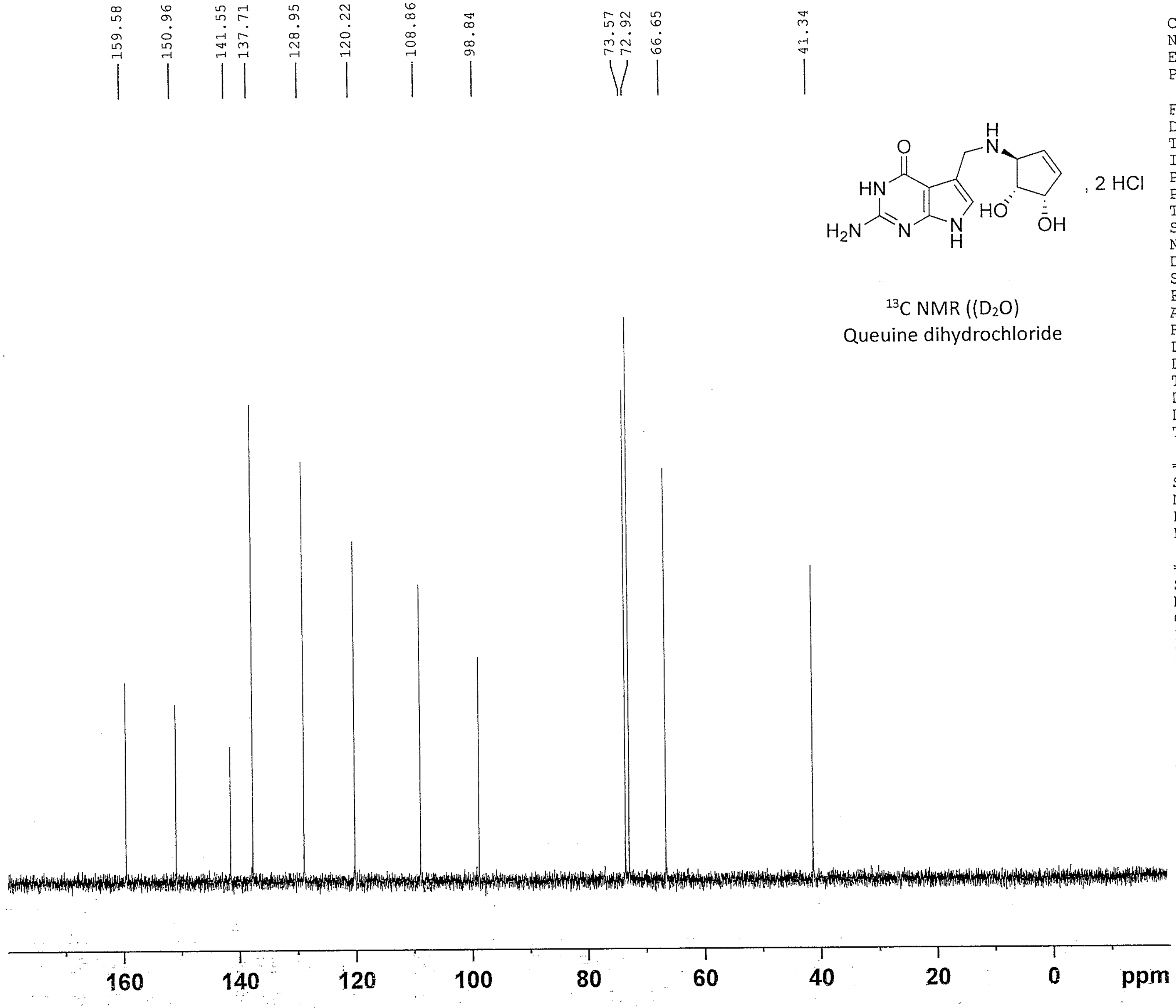

^{13}C NMR ((D_2O)
Queuine dihydrochloride

Current Data Parameters	
NAME	SN0026L5
EXPNO	10
PROCNO	1
F2 - Acquisition Parameters	
Date_	20200924
Time_	20.03
INSTRUM	spect
PROBHD	5 mm PABBO BB/
PULPROG	zgpg30
TD	65536
SOLVENT	D2O
NS	1024
DS	4
SWH	24038.461 Hz
FIDRES	0.366798 Hz
AQ	1.3631488 sec
RG	199.3
DW	20.800 usec
DE	6.50 usec
TE	295.0 K
D1	2.00000000 sec
D11	0.03000000 sec
TD0	1
======== CHANNEL f1 ========	
SFO1	100.5272687 MHz
NUC1	13C
P1	8.60 usec
PLW1	87.00000000 W
======== CHANNEL f2 ========	
SFO2	399.7515990 MHz
NUC2	1H
CPDPRG[2	waltz16
PCPD2	90.00 usec
PLW2	19.20000076 W
PLW12	0.20000000 W
PLW13	0.15552001 W
F2 - Processing parameters	
SI	32768
SF	100.5172152 MHz
WDW	EM
SSB	0
LB	1.00 Hz
GB	0
PC	1.40

SHIMADZU

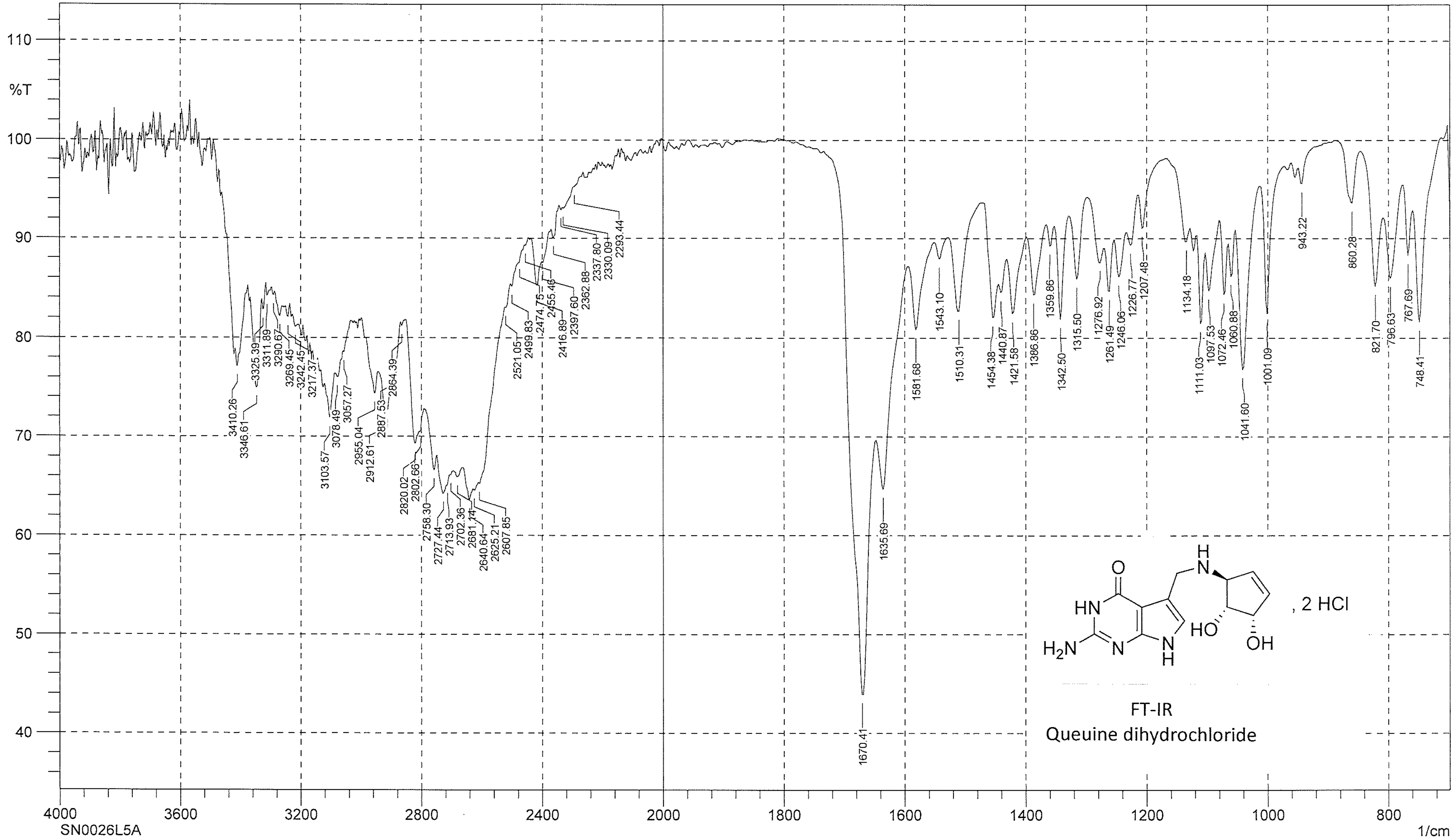

Comment;
SN0026L5

No. of Scans;
Resolution;
Apodization;

Date/Time; 23/09/2020 16:48:25
User; Administrateur

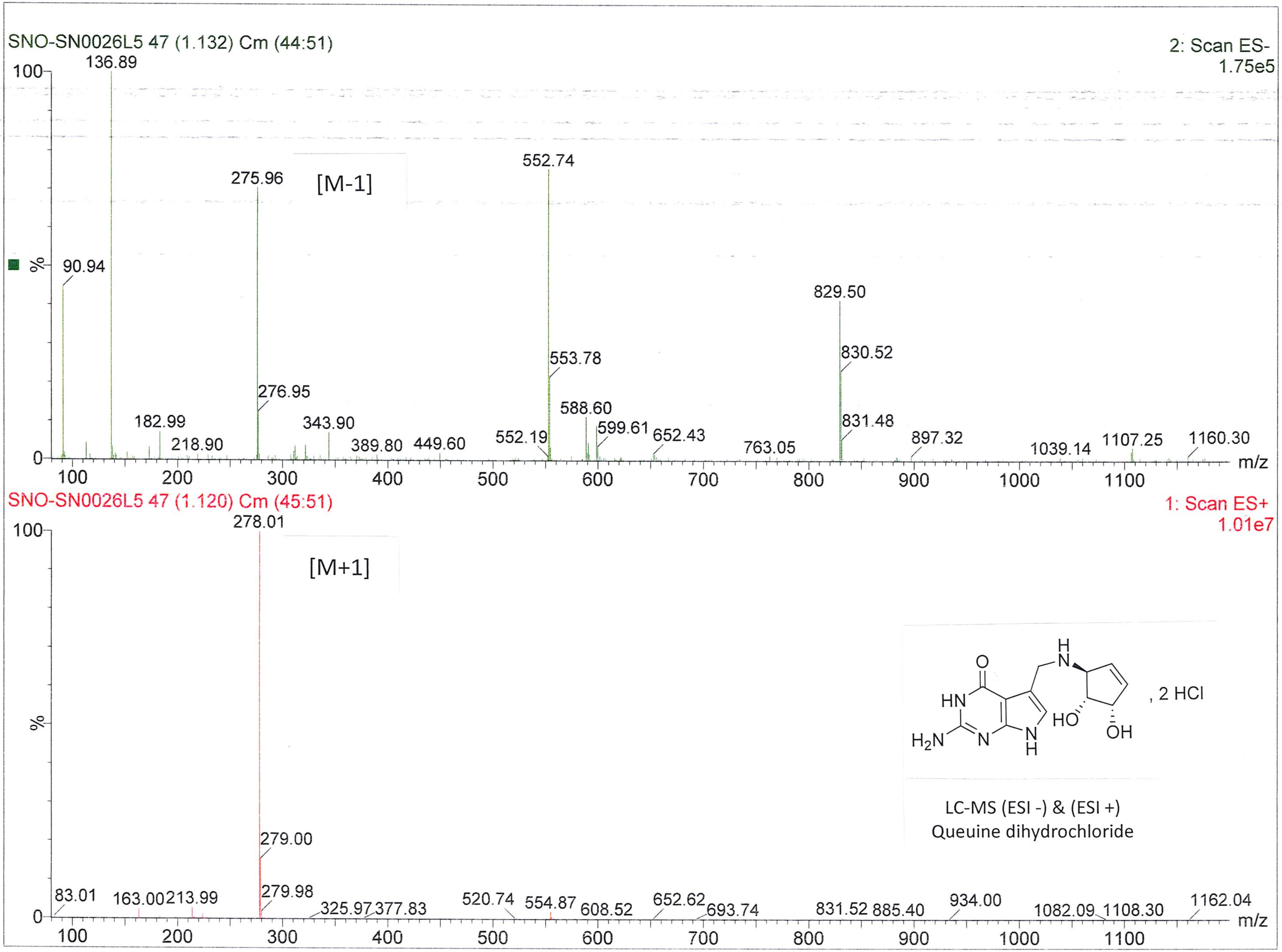

SNO-SN0026L5 47 (1.132) Cm (44:51)
2: Scan ES-
1.75e5
[M-1]
136.89
90.94
182.99
218.90
275.96
276.95
343.90
389.80
449.60
552.19
552.74
553.78
588.60
599.61
652.43
763.05
829.50
830.52
831.48
897.32
1039.14
1107.25
1160.30
%
m/z
SNO-SN0026L5 47 (1.120) Cm (45:51)
1: Scan ES+
1.01e7
[M+1]
278.01
279.00
279.98
83.01
163.00
213.99
325.97
377.83
520.74
554.87
608.52
652.62
693.74
831.52
885.40
934.00
1082.09
1108.30
1162.04
, 2 HCl
LC-MS (ESI -) & (ESI +)
Queuine dihydrochloride

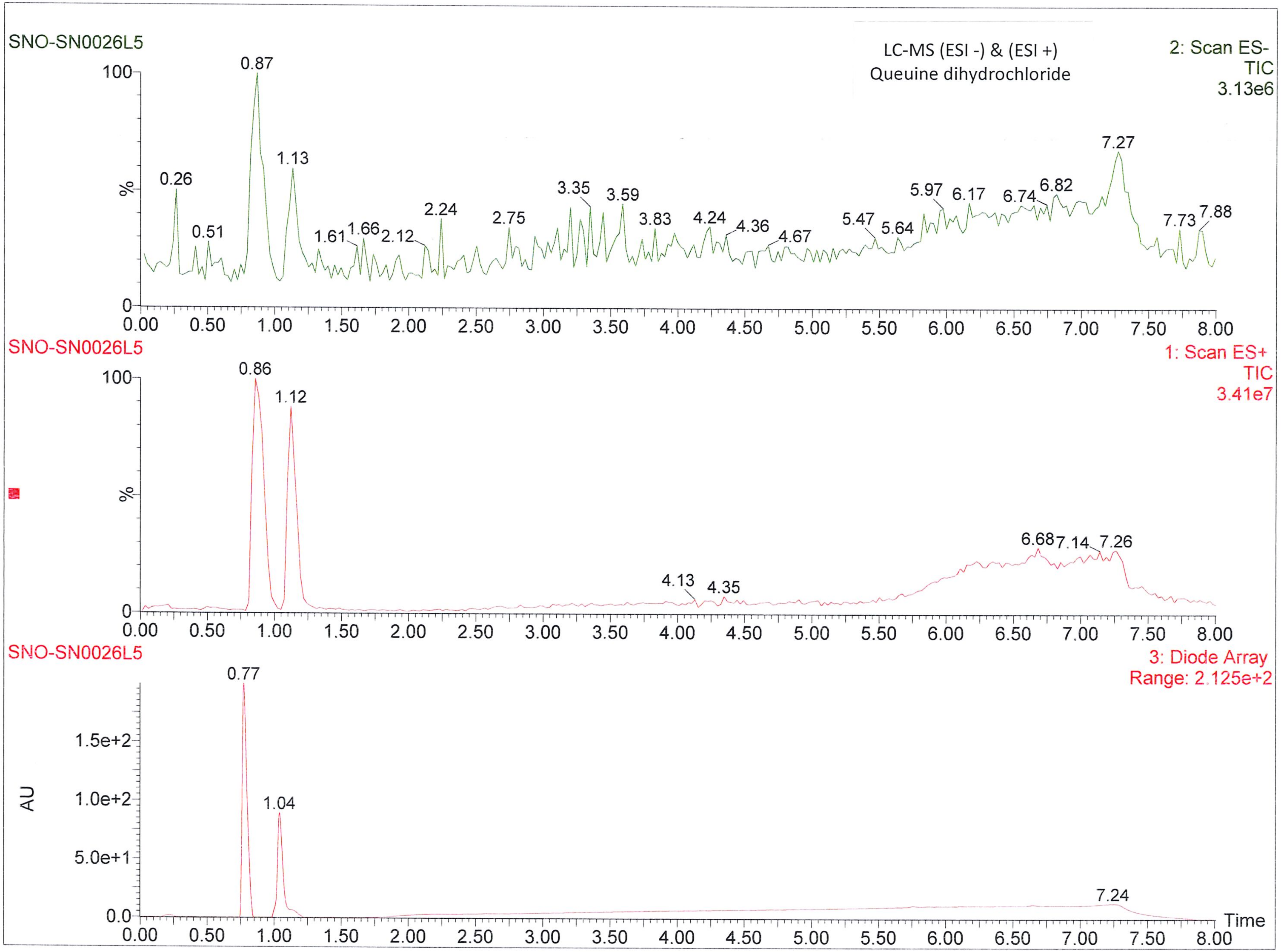

LC-MS (ESI -) & (ESI +)
Queuine dihydrochloride
SNO-SN0026L5
2: Scan ES-
TIC
3.13e6
100
%
0
0.26
0.51
0.87
1.13
1.61
1.66
2.12
2.24
2.75
3.35
3.59
3.83
4.24
4.36
4.67
5.47
5.64
5.97
6.17
6.74
6.82
7.27
7.73
7.88
0.00 0.50 1.00 1.50 2.00 2.50 3.00 3.50 4.00 4.50 5.00 5.50 6.00 6.50 7.00 7.50 8.00
SNO-SN0026L5
1: Scan ES+
TIC
3.41e7
100
%
0
0.86
1.12
4.13
4.35
6.68
7.14
7.26
0.00 0.50 1.00 1.50 2.00 2.50 3.00 3.50 4.00 4.50 5.00 5.50 6.00 6.50 7.00 7.50 8.00
SNO-SN0026L5
3: Diode Array
Range: 2.125e+2
AU
1.5e+2
1.0e+2
5.0e+1
0.0
0.77
1.04
7.24
0.00 0.50 1.00 1.50 2.00 2.50 3.00 3.50 4.00 4.50 5.00 5.50 6.00 6.50 7.00 7.50 8.00
Time

```
Title       :
Run File    : C:\Users\DG\Documents\SYNTHENOVA\Mode opératoire\SN0026 et SN0081\SN0026L5\SN0026L5.run
Method File : c:\users\dg\documents\synthenova\mode opératoire\sn0026 et sn0081\sn002615\sn002615-2.mth
Sample ID   : Manual Sample

Injection Date: 23/09/2020 17:32    Calculation Date: 27/10/2020 17:06

Operator   : dG                     Detector Type: 0800 (1 Volt)
Workstation: HDD                    Bus Address  : 81
Instrument : Instrument #1          Sample Rate  : 10.00 Hz
Channel    : 2 = 2 1                Run Time     : 10.002 min

** LC Workstation (Demo) Version 6.41 ** 05000-31C0-FA5-10A1 **

Chart Speed =    2.17 cm/min   Attenuation = 2185         Zero Offset = 2%
Start Time  =   0.000   min     End Time    = 10.002  min   Min / Tick  = 1.00
```

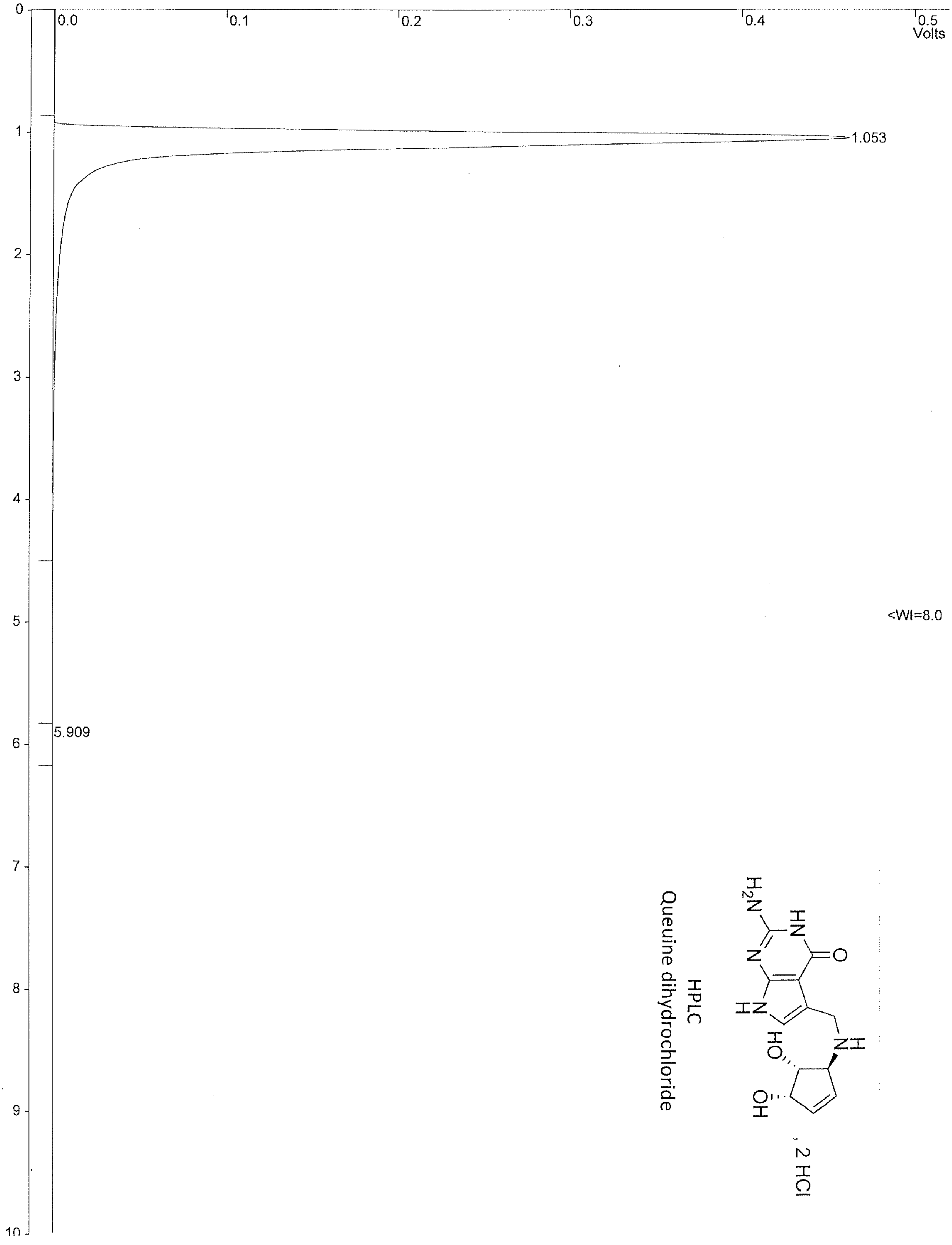

HPLC
Queuine dihydrochloride

HPLC

Queuine dihydrochloride

Title :
Run File : C:\Users\DG\Documents\SYNTHENOVA\Mode opératoire\SN0026 et SN0081\SN0026L5\SN0026L5.run
Method File : c:\users\dg\documents\synthenova\mode opératoire\sn0026 et sn0081\sn0026l5\sn0026l5-2.mth
Sample ID : Manual Sample

Injection Date: 23/09/2020 17:32   Calculation Date: 27/10/2020 17:06

Operator : dG   Detector Type: 0800 (1 Volt)
Workstation: HDD   Bus Address : 81
Instrument : Instrument #1   Sample Rate : 10.00 Hz
Channel : 2 = 2 1   Run Time : 10.002 min

** LC Workstation

Run Mode : Analysis
Peak Measurement: Peak Area
Calculation Type: Percent

Peak No.	Peak Name	Result ()	Ret. Time (min)	Time Offset (min)	Area (counts)	Sep. Code	Width 1/2 (sec)	Status Codes
1		99.9932	1.053	0.000	4626170	BB	8.0	
2		0.0068	5.909	0.000	315	BB	8.8	
	Totals:	100.0000		0.000	4626485			

Total Unidentified Counts : 4626485 counts

Detected Peaks: 2   Rejected Peaks: 0   Identified Peaks: 0

Multiplier: 1   Divisor: 1   Unidentified Peak Factor: 0

Baseline Offset: -1 microVolts   LSB: 1 microVolts

Noise (used): 41 microVolts - monitored before this run

Manual injection

****
