# Supplementary material for: Queuine, a bacterial-derived hypermodified nucleobase, shows protection in in vitro models of neurodegeneration
Source: PLoS One. 2021 Aug 11;16(8):e0253216. doi: 10.1371/journal.pone.0253216 (PMC8357117; doi:10.1371/journal.pone.0253216)
Supplement: Attachment — Submitted filename: Response to Reviewers.docx [file pone.0253216.s005.docx]

**Response to the academic editor and reviewers**

The academic editor and reviewers’ comments highlighted several important points that needed further clarification which we have provided. We found these comments valuable and below we describe our response point by point. Academic editor and reviewers’ comments are in blue.

**Academic editor**:

1. Please ensure that your manuscript meets PLOS ONE's style requirements, including those for file naming.

As recommended, we followed PLOS ONE’s style requirements.

2. To comply with PLOS ONE submissions requirements, please ensure you have provided methods of sacrifice in the Methods section of your manuscript for all experiments.

Methods of sacrifice have been added in the Materials and Methods section for each experiment.

3. Please include both an updated Funding Statement and Competing Interests Statement in your cover letter.

Updated Funding and Competing Interests Statements have been included in the cover letter:

“The authors received no specific funding for this work. Authors [PR, LK, HG, PG, NCM, XM] are affiliated to Stellate Therapeutics. The funder provided support in the form of salaries for authors [PR, LK, HG, NCM, XM], but did not have any additional role in the study design, data collection and analysis, decision to publish, or preparation of the manuscript. The specific roles of these authors are articulated in the ‘author contributions’ section.

This affiliation does not alter our adherence to PLOS ONE policies on sharing data and materials.”

4. Please include a caption for figure 3

A caption for Figure 3 (now Figure 4) already existed and can be found on page 12 of the manuscript.

**Additional Editor Comments**:

1. Reviewer #2 said "...add a cartoon to describe what you are referring to when you state that queuine replaces guanine at the wobble position 34 of tRNAs with GUN anticodons." The Reader should see what the structure of queuine is and how it "replaces guanine at the wobble position..."

We added a figure (Figure 1) that is now part of the introduction explaining the exchange of guanine by queuine into tRNAs and showing the structure of queuine.

2. Synthetic queuine. (a) You should show a chromatogram of the compound. (b) What is its percent purity? (c) You must have performed mass spec on the synthesized queuine. What was the synthetic compound's mass as determined by mass spec? What is its theoretical mass?

Additional details about STL-101 have been added to the STL-101 synthesis paragraph in the Materials and Methods section on page 17 (see response to the major comment #1 of reviewer #1 below).

HPLC, IR, NMR and LC-MS spectra can be found in supporting information as the “queuine dihydrochloride analyses” file.

Reviewer #1:

Major comments:

1. The manuscript has very little information about the synthesis of STL-101 (results, methods or figure legends). Please describe this somewhere. For example, the referenced paper for synthesis states that additional analogues of queuine are possible. Is this an analog or the original? Was this compound dissolved in water or a solvent? If a solvent is used, which one and at what final concentration in the experiments?

Spectra of STL-101 analyses can now be found as supporting information and the paragraph “STL-101 synthesis” in the Materials and Methods page 17 now indicates the required information about synthesized queuine (STL-101):

“STL-101 is queuine dihydrochloride (C_12_H_15_N_5_O_3_ (2HCl)) with a molecular weight (MW) of 350.20 g.mol^-1^ (natural queuine has a MW of 277.28 g.mol^-1^)_._ STL-101 purity was determined at 99% by HPLC and was solubilized in water, PBS or DMSO at 10-30 mM as stock solution. Spectra related to STL-101 analysis can be found in the supporting information file entitled “queuine dihydrochloride analyses”.

The final concentration of STL-101 used is indicated on each graph.

2. As a related comment, there are no solvent controls mentioned in the manuscript that I can find. Please include in Figure 1D and in Figure 2B-D, and Figure S1 A-D. This will serve as a negative control and as a comparison to the activity observed vs. the neuronal incitor in each of these experiments.

The vehicle used in each experiment is now indicated in Materials and Methods and in Figure captions.

For Figure 1A-C (now Fig 2A-C), the vehicle used is PBS (now indicated on page 17/18 of the “Primary culture of cortical neurons and huPFFs challenge” section and Fig 2C caption on page 9).

For Figure 1D (now Fig 2D), culture medium was used as indicated on page 19 in the “Culture of mesencephalic dopaminergic neurons, STL-101 pretreatment and MPP+ injury at 10μM and immunostaining” section and in the figure caption of Fig 2D on page 9.

For Figure 2B-D (now Fig 3B-D) and S1A-D (now Fig S2A-D), STL-101 has been solubilized in DMSO and diluted in culture medium (final concentration in DMSO = 0.1%). The vehicle used for control (Ctrl) = culture medium with 0.1% DMSO as indicated in Figure 3D (page 11) and S2D (page 33) captions. This information can now also be found on page 22 of the Materials and Methods section.

For Figure S1, vehicle = culture medium as indicated in Fig S1B caption (page 33) and on page 19 of the “Culture of mesencephalic dopaminergic neurons, STL-101 and MPP+ (4μM) co-treatment, immunostaining and analysis” section of the Materials and Methods.

3. The concentration of STL-101 required for significant protection of MPP+ protection of DA neurons was much higher than that for huPFFs (1 uM vs. 0.1 uM) or the Abeta acute model. The abstract describes the utility of queuine for the facilitation of protein misfolding and prevention of protein aggregation. This concept should be further described within your dataset and compared with the MPP+ model which is certainly not considered a traditional protein misfolding model (and required much higher amounts of STL-101 for efficacy).

We performed another *in vitro* experiment using an MPP+ injury at 4μM instead of 10μM and a co-treatment with STL-101 instead of a pre-treatment of 1 day. The data can now be found in Figure S1. We obtained a similar neuroprotective effect of STL-101 (although a significant effect is seen at 3μM only probably due to the lack of pretreatment) in this model than the one previously observed (Fig 2D). In the same model, we also looked at the α-syn signal by IF since it has previously been reported that MPP+ also triggers α-syn aggregation. Indeed Callizot et al., 2019 showed that increased signal of α-syn in DA neurons corresponds to hyper-phosphorylated/aggregated α-syn. Our result (Fig S1B) shows a significant decrease in the α-syn signal in DA neurons when the cells are treated with STL-101 at 1μM and above.

In order to fully address comment #3, we added the following text in the discussion on page 13 of the manuscript:

“We note that STL-101 concentration required for significant protection is higher with the MPP+ model than the other models (1µM vs. 0.1-0.3µM respectively). STL-101 was added 4 days prior huPFFs exposure, which might contribute to a better protection than no pretreatment or 1 day pretreatment before injury (MPP+ or Aβ_1-42_). It is also possible that the mitochondrial stress induced by MPP+ alters STL-101 function (e.g., oxidation of the molecule, reduction of cellular intake) which would require higher amount of the molecule to reach protection. Interestingly, significant decrease of α-syn aggregation in the MPP+ model is seen when STL-101 is added without pretreatment at 1µM and above while significant neuronal survival is seen with STL-101 at 3µM. This indicates that some MPP+-induced phenotypes can be rescued by STL-101 near physiological concentrations (α-syn aggregation) while others require STL-101 at pharmacological concentrations (cell survival). This could also indicate that STL-101 treatment reduces/prevents α-syn aggregation before affecting cell survival. It is possible that by optimizing mRNA translation and preventing misfolding of newly synthesized protein, STL-101 delays and/or prevents protein aggregation (in a seeding model such as huPFFs as well as in a model where aggregation might be triggered by mitochondrial dysfunction (e.g., MPP+ intoxication)) that needs to reach a certain level before it can prevent neuronal loss.”

Minor comments:

1. This manuscript has the potential to attract wide-spread readership. However, many readers will not be familiar with GUN anticodons and wobble position replacement. Therefore, as part of the introduction, could you please add a cartoon to describe what you are referring to when you state that queuine replaces guanine at the wobble position 34 of tRNAs with GUN anticodons?

We have now added a Figure (Figure 1) in the introduction describing queuine integration into tRNAs.

2. Similarly, I muddled through reading the terms queuine vs. queosine. Please add something of emphasis to the writing (or in an introductory figure) to help the time-crunched reader remember the difference the first time they read it.

Figure 1 also states what is queuine and what is queuosine.

3. What program was used to calculate statistics? This should be included in the Stats section.

GraphPad Prism was used to perform all the stats and this information is now in the “Statistical analysis” section on page 26.

4. In figure 1C, these data are described in micromolar. However, in figure 1B (legend and results section), the concentration here is described in nanomolar. While the conversion is easy enough, it is inconsistent. Additionally, in Figure 1C, there is a 0 micromolar data point. What was the solvent?

The concentration has been changed to µM in the caption of Figure 2B (former Figure 1B). As now indicated in the caption of Figure 2C, the solvent is PBS.

5. Similarly, in Figure 2, there is a discrepancy between nanomolar and micromolar terminology.

µM is now used in the caption of Figure 3 (former Figure 2).

6. In the first sentence of the discussion, the word “remarkable” seems a bit overboard and could probably be eliminated without changing the sentence or meaning of your nice work, at all. Moreover, stating in this same sentence that queuine is exclusively produced by bacteria and causes neurodegeneration seems misleading, since, in this study, the STL-101 was made in a lab. Perhaps you could break these thoughts into two different sentences to remind the reader that naturally, queuine is exclusively made by bacteria, but in this study it was chemically synthesized

As suggested, the first sentence of the discussion is now:

“Here we have shown protective properties of chemically synthesized queuine, a molecule naturally exclusively produced by bacteria, in several *in vitro* models of proteinopathies and neurodegeneration.”
